# Climate and agronomy, not genetics, underpin recent maize yield gains in favorable environments

**DOI:** 10.1073/pnas.2113629119

**Published:** 2022-01-18

**Authors:** Gonzalo Rizzo, Juan Pablo Monzon, Fatima A. Tenorio, Réka Howard, Kenneth G. Cassman, Patricio Grassini

**Affiliations:** ^a^Department of Agronomy and Horticulture, University of Nebraska–Lincoln, Lincoln, NE 68583-0915;; ^b^Department of Statistics, University of Nebraska–Lincoln, Lincoln, NE 68583-0963

**Keywords:** climate, agronomy, genetics, yield gain, yield potential

## Abstract

Quantitative understanding of factors driving yield increases of major food crops is essential for effective prioritization of research and development. Yet previous estimates had limitations in distinguishing among contributing factors such as changing climate and new agronomic and genetic technologies. Here, we distinguished the separate contribution of these factors to yield advance using an extensive database collected from the largest irrigated maize-production domain in the world located in Nebraska (United States) during the 2005-to-2018 period. We found that 48% of the yield gain was associated with a decadal climate trend, 39% with agronomic improvements, and, by difference, only 13% with improvement in genetic yield potential. The fact that these findings were so different from most previous studies, which gave much-greater weight to genetic yield potential improvement, gives urgency to the need to reevaluate contributions to yield advances for all major food crops to help guide future investments in research and development to achieve sustainable global food security. If genetic progress in yield potential is also slowing in other environments and crops, future crop-yield gains will increasingly rely on improved agronomic practices.

## Significance

After accounting for the effect of climate and improvements in agronomic management, we found the contribution of genetic technologies to increasing maize yield potential in favorable environments was substantially smaller than reported in previous studies. If genetic progress in yield potential is slowing in other environments and for other crops as well, future production gains will increasingly rely on yield gains from improved agronomic practices and/or increasing crop intensity where possible.

Demographic, economic, and dietary trends will require substantial increases in yields of staple grain crops on existing production area to avoid conversion of natural ecosystems to farmland (1, 2). However, there is evidence of slowdown in yield gains and even yield plateaus in some high-yield cropping systems of the world, including rice in China and California (United States) and wheat in northwestern Europe (3–5). Hence, understanding the factors driving crop yield gains during recent decades is essential to inform future public- and private-sector investments in agricultural research and development to achieve adequate rates of yield gain.

Past gains in farm yield resulted from adoption of improved crop- and soil-management practices (hereafter called improved agronomic practices), better cultivars and hybrid seed (hereafter called genetic technologies) that have greater yield potential, and their interactive effects (6–9). Here, we define yield potential as the yield of a well-adapted cultivar as determined by atmospheric carbon dioxide (CO_2_), temperature, and solar radiation in absence of limitations from water, nutrients, weeds, pathogens, and insect pests (10). As a reference, the grain yield that a competent grower can achieve with unrestricted irrigation supply using the best-available agronomic and genetic technologies typically gives 80% of yield potential (11, 12). Short (decadal)- and longer-term (century) climate trends and changes in atmospheric CO_2_ level over time also influence farm yield trends.

The United States produces ca. one-third of global maize production. Average US maize yields have increased steadily over past decades due to breeding and improved agronomic management (e.g., refs. 6 and 12–14). There has been comparably less research about the progress made toward increasing maize yield potential, that is, crop yields under stress-free conditions and the weather conditions typical of a region. Based on analysis of reported yields from contest-winning fields in Nebraska, Duvick and Cassman (14) concluded that yield potential for irrigated maize has remained unchanged during the 1983-to-1997 period. Consistent with this finding, Grassini et al. (15) found, for the same region, evidence of an incipient plateau in farm yields in high-yielding irrigated maize systems in which average yield was approaching yield potential (ca. 80% of yield potential).

In contrast, most other published reports assessing maize yield gains in favorable production environments estimated more-rapid rates of increase in yield potential, attributing most of the increase to improvements in genetic technologies. In these studies, vintage sets of hybrids were grown in today’s environment, ensuring proper control of biotic stresses, with the slope of the relationship between grain yield and year of release of each hybrid taken as an estimate of progress in genetic yield potential (16). For example, based on a comparison of maize hybrids released between 1963 and 2011 grown at their optimal plant density in well-watered, fertilized, and protected field experiments, Messina et al. (17), Cooper et al. (18), and Smith et al. (7) reported that yield potential has increased at a rate ranging from 81 to 95 kg ha^−1^ y^−1^ (0.60 to 0.80% per annum [p.a.] when expressed as compound annual growth rates). Following a similar experimental approach, Di Matteo et al. (19) reported that yield potential has increased at a rate of 107 kg ha^−1^ y^−1^ (0.83% p.a.). These rates of gain in yield potential are consistent with those reported by others for rainfed production environments during crop seasons with favorable weather conditions (e.g., ref. 20). However, both Smith et al. (7) and Di Matteo et al. (19) admitted that a less-optimistic interpretation of their data is possible (i.e., much-smaller or even nil gain in genetic yield potential) when the analysis was restricted to the hybrids released over the last 20 y of their time series. In addition, the experimental design used in these studies had an inherent bias as “old” hybrids selected decades ago were compared against more-recently released hybrids in trials conducted in today’s environment. As a result of the breeding selection process, modern cultivars were more adapted to current management, climate, and soil properties compared with older cultivars, which could have led to an overestimation in the rate of yield-potential gain over time even in the absence of biotic stresses (21–24).

Analysis of farm yield trends in high-yield irrigated environments can help identify factors responsible for increase in yield potential over time. Interpreting yield trends is difficult, due to the confounding effects of climate, management practices, genetics, and their interactions. A number of previous studies have used crop-modeling to remove the climate effect and isolate the technology-driven yield trend (i.e., yield trend due to adoption of genetic and agronomic technologies). For example, Bell and Fischer (25) investigated yield trends of irrigated wheat in the Yaqui Valley (Mexico) during the 1968-to-1990 period, assuming constant agronomy and genetics for isolating the effect of changing climate on yield potential. These authors found a decrease in climate-driven yield potential over time associated with an upward trend in temperature. Once the climate effect was removed from the analysis, the technology-driven rate of yield gain increased from 57 to 103 kg ha^−1^ y^−1^. Similarly, Hochman et al. (26) showed that the technology-driven yield gain for wheat in Australia from 1990 to 2015 has been underestimated due to a decline in seasonal precipitation over the same time period. In the case of temperate maize in the United States, Tollenaar et al. (27) and Ortiz-Bobea and Tack (28) also found that climate has influenced recent gains in farm yields. Even when the climate effect can be estimated in these previous studies, it remains difficult to separate the contributions of genetic and agronomic technologies to the total yield gain.

Here, we use an approach that combines farmer-reported data and crop-modeling to identify the drivers of irrigated maize yield gain in the largest irrigated maize-production domain in the world, in which farmers consistently use best-available hybrids and achieve maize yields above 12 Mg ha^−1^. The analysis is based on data collected annually from ca. 3,000 irrigated maize fields located in three regions in Nebraska over 14 y (2005 to 2018), together with a well-validated crop model, good-quality data on climate, and detailed data on crop-management practices (*SI Appendix*, Figs. S1 and S2 and Table S1). The high-input, high-yield irrigated maize-production system in Nebraska provides an ideal context to quantify the relative contribution of agronomic and genetic technologies to yield gain and to estimate changes in yield potential over time.

## Results

### Yield Gain as Driven by Climate and New Technologies.

**Fig. 1.**
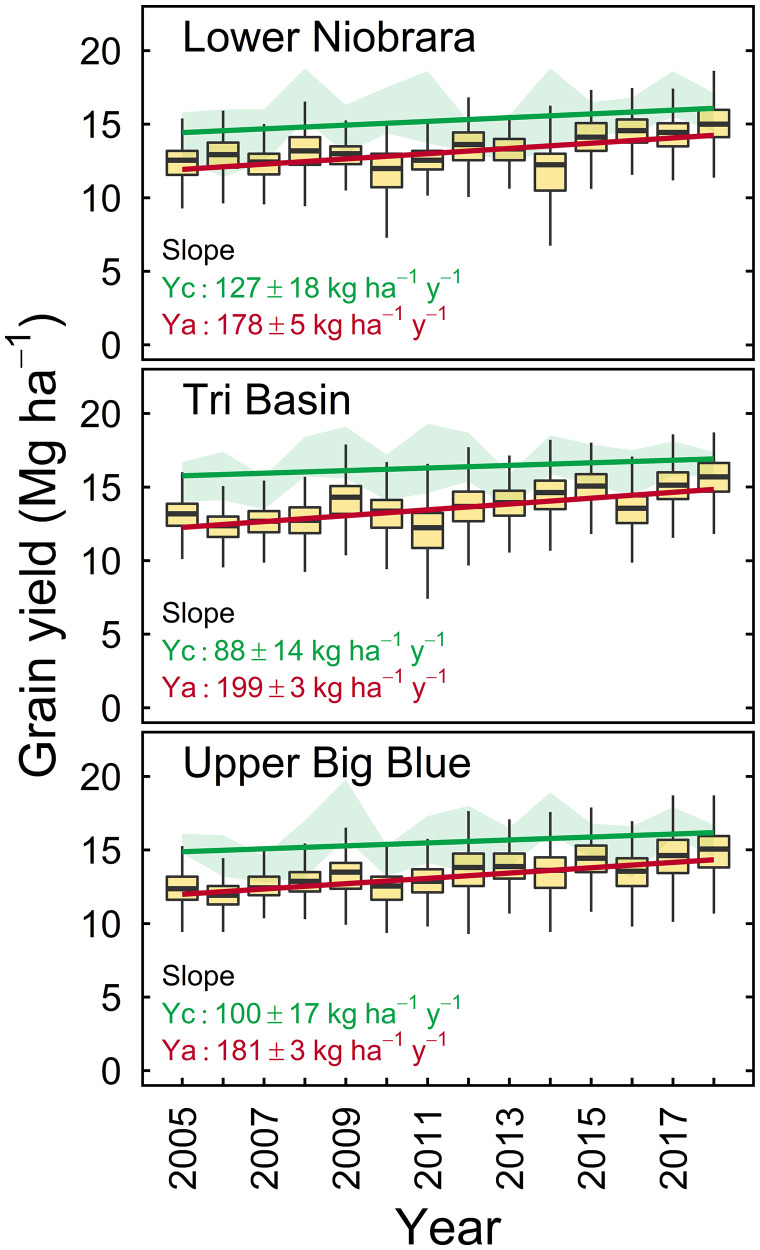
Simulated climate-driven yield potential (Yc) and average farm yield (Ya) for irrigated maize in three regions in Nebraska: Lower Niobrara (*Top*), Tri-Basin (*Middle*), and Upper Big Blue (*Bottom*). The green shadow indicates the range of simulated Yc across nine combinations of sowing date and hybrid maturity for each year. Box plots show Ya, with boxes delimiting the 25th and 75th percentiles and lines indicating fifth and 95th percentiles; the horizontal line within each box represents the median. Also shown are fitted linear-regression models for Yc (green) and Ya (red) and their associated slopes (± SEs). Slopes of fitted regression models were statistically different from zero in all cases (*P* < 0.01).

**Fig. 2.**
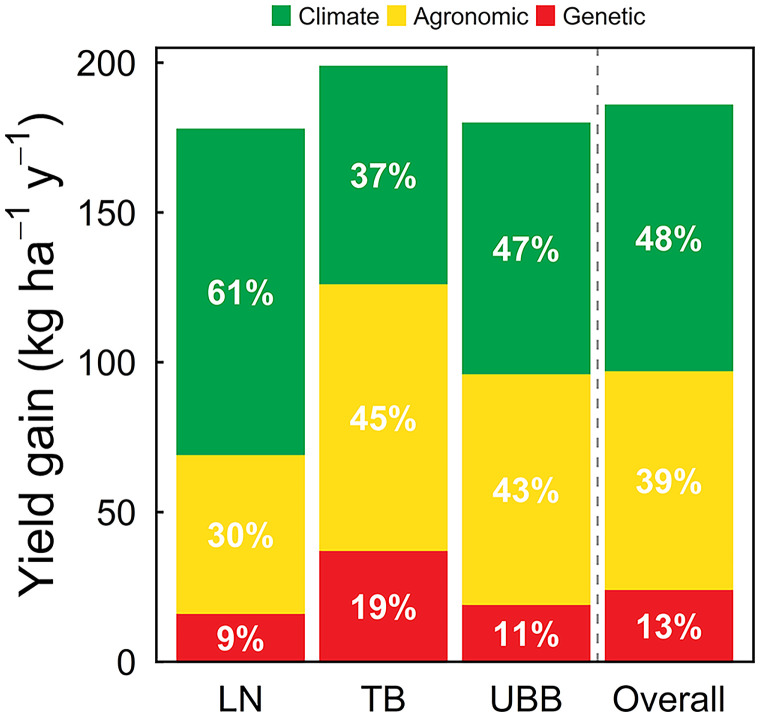
Total yield gain and contribution from changes in climate and adoption of agronomic and genetic technologies for each region: Lower Niobrara (LN), Tri-Basin (TB), and Upper Big Blue (UBB). Also shown are the averages across the three regions. Numbers inside bars indicate the relative contribution of climate (green), agronomic management (yellow), and genetic improvement (red) to the total yield gain.

Average (2005 through 2018) simulated yield potential was 15.7 Mg ha^−1^, ranging from 15.3 to 16.3 Mg ha^−1^ across the three regions in our study. Average farm yield was 13.2 Mg ha^−1^, following the same trend in yield potential across regions. Annual average climate-driven yield potential and average farm yield were both relatively stable over time (interannual coefficients of variation = 5% and 12%, respectively), because irrigation buffered against year-to-year variation in seasonal precipitation. Increase in yield potential due to climate contributed 89 kg ha^−1^ y^−1^ when averaged across the three regions (*P* < 0.01) (Figs. 1 and 2 and *SI Appendix*, Table S2). Overall, the climate-driven yield gain represented 48% of the total gain during that period, which means that 52% of the observed yield gain was driven by adoption of improved genetic and agronomic technologies. Across the three regions, the “true” technological yield gains (i.e., unrelated to climate) averaged 97 kg ha^−1^ y^−1^ (or 0.68% p.a.), ranging from 69 kg ha^−1^ y^−1^ (0.49% p.a.) to 126 kg ha^−1^ y^−1^ (0.87% p.a.).

### Drivers of Climate and Technological Yield Gains.

**Fig. 3.**
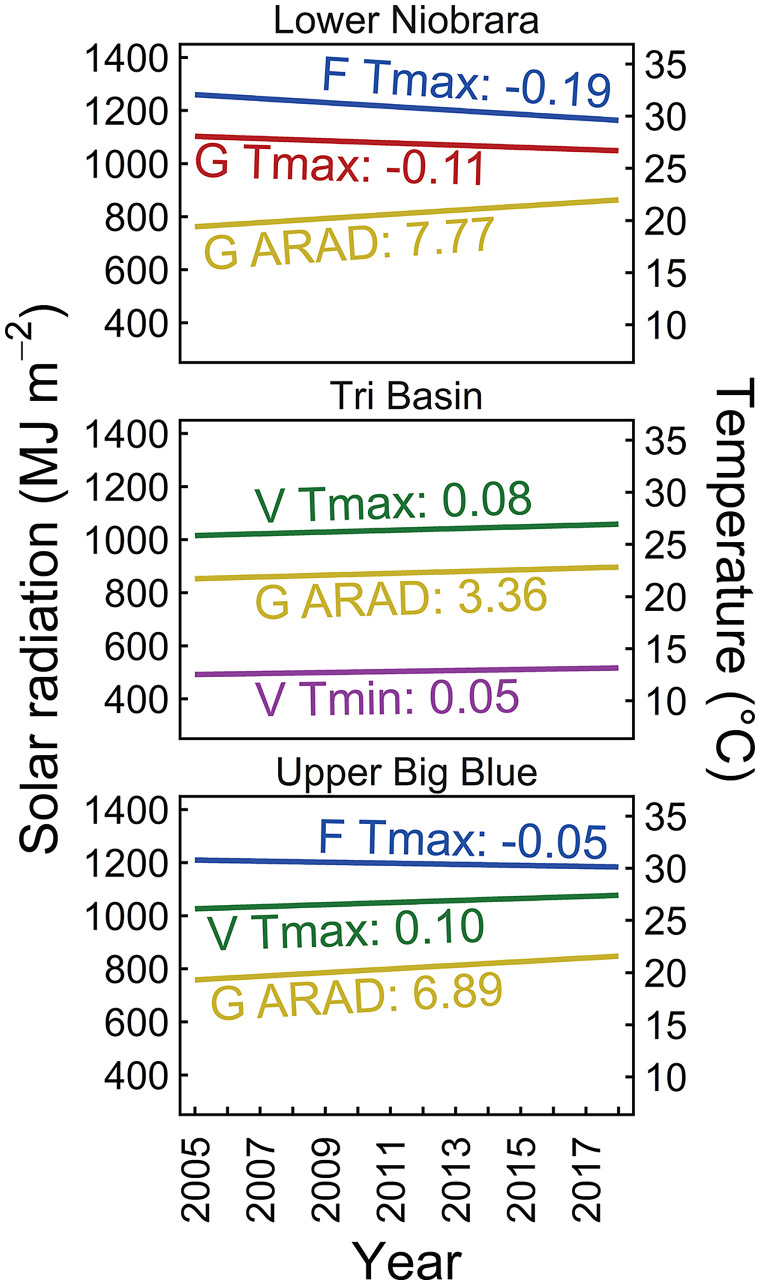
Temporal trends in climate variables during the 2005-to-2018 period in three regions: Lower Niobrara, Tri-Basin, and Upper Big Blue. Only shown are trends that were statistically significant (*P* < 0.01). Lines represent the fitted linear-regression models. Also shown are slopes of the fitted linear-regression models for accumulated solar radiation (ARAD), average maximum temperature (Tmax), and minimum temperature (Tmin) computed for three crop phases: vegetative (V), flowering (F), and gran filling (G).

Because yield potential is determined by temperature and solar radiation during the growing season, the significant upward trend in yield potential was therefore associated with a decadal trend of increasingly more-favorable weather during the study period. We found an upward trend in cumulative solar radiation during grain-filling over the study period from 2005 to 2018 (*P <* 0.01), which largely explained the climate-driven yield gain for irrigated maize (Fig. 3). Notably, the higher cumulative solar radiation over time was caused by a steady, increased duration of the grain-filling phase (*P <* 0.01) associated with cooler temperatures without a detectable increase in daily solar radiation (*P >* 0.25). Depending on region, longer grain-filling duration was driven by a downward trend in T_max_ during flowering and/or grain-filling and/or an upward trend in T_min_ and/or T_max_ during the vegetative phase leading to a shorter duration of the vegetative phase. Shorter vegetative phase also led to a shift of silking and grain-filling earlier toward the summer solstice and, therefore, more-favorable weather conditions for grain-setting and -filling (e.g., lower temperature and higher daily incident radiation). Also included in the climate-driven gain was a small yield gain (6 kg ha^−1^ y^−1^) driven by an increase in atmospheric CO_2_ concentration (+30 ppm) during the study period.

Analysis of the management practices around year 2005 versus 2018 revealed a number of changes in agronomic management over time (Table 1). For example, average seeding rate was 10% higher in 2018 compared with 2005, while the average N fertilizer rate increased 20% during that time. A substantial increase in the proportion of fields under conservation tillage took place from about one-third of the fields in 2005 to near 80% of fields in 2018. Likewise, in-season application of fungicide and/or insecticide increased markedly from ca. one-quarter (2005) to two-thirds of the fields (2018). There was also a consistent 5% increase in the proportion of fields following a maize–soybean rotation instead of continuous maize. Changes in management practices were consistent across regions (*SI Appendix*, Table S3).

**Table 1.** Changes in management practices between 2005 and 2018 based on survey data collected from a subset of 268 farmers across the three regions

Management practice	Average		Change	Yield gain
	2005	2018		kg ha^−1^ y^−1^
Sowing date (DOY)	120	121	+1	
Seeding rate (seed m^−2^)	7.4	8.0	+0.6*	+28
Cultivar relative maturity (d)	112	112	nil	
Conservation tillage (% fields)	33	83	+50*	−13
Rotation with soybean (% fields)	48	54	+6*	+2
Foliar fungicide and/or insecticide (% fields)	27	61	+34*	+7
Grazing prior crop stover (% fields)	43	43	nil	
Applied N fertilizer (kg N ha^−1^)	187	220	+33*	+50

Averages for each year and the difference between 2018 and 2005 values are shown. Estimated annual yield gain associated with changes in individual management practices are also shown (*SI Appendix*, Table S4). Changes in management practices are shown separately for each of the three regions in *SI Appendix*, Table S3. Asterisks indicate statistically significant difference (*P* < 0.05) using *t* test or χ^2^ test (for variables with normal or binomial distribution, respectively).

Our study estimated the magnitude of contribution from the observed changes in agronomic technologies. Increased rates of applied N fertilizer and higher seeding rates, together with changes in tillage, crop rotation, and application of foliar fungicide and/or insecticide, contributed 73 kg ha^−1^ y^−1^ to total yield gain (average: 0.51% p.a.), ranging from 53 to 89 kg ha^−1^ y^−1^, representing 75% of the technology-driven yield gain (Fig. 2 and *SI Appendix*, Table S5). A small yield penalty was associated with the use of conservation tillage in irrigated maize (range: −6 to −15 kg ha^−1^ y^−1^), which was offset by the positive yield effect associated with changes in the other management practices. The remaining portion of the technology-driven yield gain provided an estimate of impact from genetic gain in yield potential, which averaged 24 kg ha^−1^ y^−1^ across regions (average: 0.17% p.a.), representing 23% of the technology-driven yield gain and only 13% of the total yield gain (Fig. 2 and *SI Appendix*, Table S5).

## Discussion

Using a large, farmer-reported database representing a substantial proportion of irrigated US maize production, high-quality data on weather and management practices, and robust crop simulation, we found that nearly half the yield gain from 2005 to 2018 was attributable to a favorable climate trend during this period. In addition, our analysis estimates a technology-driven yield gain associated with widespread adoption of agronomic technologies and improved hybrids, accounting for the other half of total yield gain. We view this finding with optimism, because others have found strong evidence of yield plateaus (15) and negative impact of climate change on US maize yield (29–31). However, from a global perspective, the technology-driven yield gain estimated for irrigated maize in Nebraska is only half of the global rate of yield gain for maize (0.68 versus 1.27% p.a.) during the same time period (2005 through 2018) (32). This finding is consistent with the notion that yield gains become more difficult to achieve in cropping systems in which average farm yield is near yield potential (12), as is the case for irrigated maize in the United States.

Genetic improvement of maize yield potential only accounted for 13% of the total yield gain. The contribution of genetic technologies found here (0.17% p.a.) is three to four times smaller than that reported in previous studies for well-watered maize grown at optimal plant density, ranging from 0.60 to 0.83% p.a. (7, 17, 18). Subtle changes in seed protein concentration, phenology, canopy architecture, and dry matter partitioning to grain are included in our estimate of yield gain from genetic improvement (6, 14, 33, 34). Our estimate of genetic yield potential gain may be inflated, as it includes contributions from insect- and herbicide-resistance traits, which may have helped to increase farm yields but not yield potential per se.

Our study shows that previous predictions of sharp increases in maize yield potential (2 to 3.6% p.a.) with the advent of biotechnology and molecular techniques have fallen short of reality (e.g., refs. 34–39). Indeed, we found the rate of genetic gain in maize yield potential to be less than a third of the yield gain due to management (0.17 versus 0.51% p.a.), suggesting that the rate of yield increase of maize grown in favorable environments will slow markedly over coming decades. This finding is of particular concern, considering that investment in maize genetic-improvement research and development in the public and private sector has been considerably larger than that for other crops. If similar trends are occurring in the other major staple food grains, as it has been reported for rice and wheat (22, 40), opportunities to increase yields on existing farmland in irrigated and favorable rainfed environments will more likely come from increased cropping intensity (more crops per year) rather than higher yields per crop and especially so when global warming trends are considered (1, 8, 41, 42).

Our study provides an alternative approach to estimate technological yield gains based on data collected from farmer maize fields in a high-yielding irrigated environment. We recognize that by calculating the genetic yield gain by difference, it can accumulate errors derived from estimation of climatic and agronomic yield gains. Even in that case, we do not foresee a consistent bias in one direction (i.e., overestimation or underestimation). In addition, our study is subject to a number of uncertainties. For example, our estimate of climate-driven yield potential did not consider changes in tropospheric ozone level (43). Likewise, the approach followed here can be partly confounded by interactive effects among management practices, cultivars, and climate (44). For example, better performance of modern hybrids at higher optimal plant density could be attributed to better genetic tolerance to crowding (34). Regardless, our approach provides an independent measure of separate contributions to crop-yield gains from widespread farmer adoption of management and genetic technologies. The approach that we used complements previous studies based on evaluation of an array of historical cultivars grown in replicated field plots in today’s environments, which, as mentioned previously, are subjected to a number of important methodological limitations. Our study also suggests caution in using recent crop yield trends to estimate future food production potential without correction for recent climate trends, because doing so can give misleading estimates of global capacity to meet future food demand on existing cropland. Likewise, we note that the rate of yield-potential gain from adoption of new technologies as estimated here (0.68% p.a.) is below that needed to meet maize demand on existing area by 2050 (ca. 1% p.a.; ref. 57. If these trends persist over the long term, future production gains will rely on increasing yields in areas where current yields are well below their potential or from further expansion of cropland area at expense of natural ecosystems, highlighting the importance of raising crop yield potential to meet future food demand and reduce associated land and water requirements.

## Methods

### Study Area and Farmer Database.

Nebraska has the largest share of maize irrigated area in the United States (ca. 54%), with ca. 2.1 M ha sown to irrigated maize (45). Nebraska is divided into 23 Natural Resources Districts (NRDs; https://www.nrdnet.org), each NRD serving as a government entity authorized to establish regulations to conserve water and soil resource quality and quantity (46, 47). Every year, NRDs require farmers with fields located within their boundaries to report field-level data on yield and applied inputs. For our study, we used data collected by three NRDs: Lower Niobrara, Tri-Basin, and Upper Big Blue, which were referred as “regions” in the main text for simplicity (*SI Appendix*, Fig. S1 and Table S1). The three regions portrayed well the range in climate and soils across the irrigated maize area within Nebraska. Using a spatial framework that delineates biophysical domains based on similarity in climate and soils (48), we determined that selected study areas were located in environments that account for ca. 70% of the irrigated maize area in Nebraska. Average 14-y (2005 to 2018) irrigated maize yield across the three regions (13.2 Mg ha^−1^) was slightly higher (+6%) than statewide NE average for irrigated maize over the same time period (12.4 Mg ha^−1^), indicating that the surveyed fields were representative of the most-productive irrigated environments within state. Farmers’ average hybrid turnover was around 3 y. Some good hybrids may have lasted 5 to 6 y before they were replaced by similar, better ones. Farmers had access to a multitude of new hybrids every year and by careful evaluation within and among their fields, they adopted those showing overall better-yield performance. Details on the database were provided elsewhere (49, 50).

Field-level data were collected over 14 y (2005 to 2018), which was of sufficient duration to account for interannual variability in weather variables influencing yield potential, such as solar radiation and temperature (*SI Appendix*, Fig. S2). Annual data reported for each field included field location, maize grain yield (at standard moisture content of 155 g H_2_O kg^−1^ grain), N fertilizer rate, irrigation amount, and management practices such as previous crop and irrigation method (pivot or surface irrigation). Only pivot-irrigated fields were considered for our study as surface (flood) irrigation accounts for a small fraction of irrigated maize area in NE (ca. 14%), and its area has steadily declined over decades (51). We only included fields sown with maize after a maize or soybean crop the previous year because the majority (>85%) of maize across the US Corn Belt is grown either as continuous maize or in a maize–soybean rotation (49). Hence, for our study, we considered maize yield data for all years in the case of fields following continuous maize or only for the maize phase of the crop sequence in fields following maize–soybean rotation. Our study only included fields sown with maize for grain; other maize fields sown for seed production or silage were excluded, because they represent a small fraction of the total maize area. The final database used for the analysis contained a total of 41,147 field-year observations, with an average of 480, 1,405, and 1,047 reporting fields per year in Lower Niobrara, Tri-Basin, and Upper Big Blue, respectively.

### Simulation of Climate-Driven Yield Potential.

In the present study, the climate-driven yield potential was simulated using the Hybrid-Maize crop model (52–55). Hybrid-Maize is a process-oriented model that simulates maize development and growth on a daily basis under growth conditions without limitations from nutrient deficiencies or toxicities or from insect pests, diseases, and weeds. It features temperature-driven maize development, vertical canopy integration of photosynthesis, organ-specific growth respiration, and temperature-sensitive maintenance respiration. In the Hybrid-Maize crop model, the intercepted, photosynthetically active radiation and its corresponding CO_2_ assimilation are computed for each layer in the canopy, and then, total gross assimilation is obtained by integration over all canopy layers. Grain number is determined by the crop-growth rate around silking, and grain-filling rate is modulated by assimilate availability (from net assimilation and mobilization from stem and leaf) and temperature. The Hybrid-Maize model has been satisfactorily evaluated on its ability to reproduce measured yields in well-managed irrigated maize crops in which yield limiting and reducing factors were minimized, showing a root mean square error of 1 Mg ha^−1^, which represented 4% of the average observed yield (55). Hybrid-Maize does not account for the effect of air vapor pressure deficit, percentage of diffuse radiation, and ozone levels on crop photosynthesis.

We retrieved daily measured weather data from meteorological stations managed by the High Plains Regional Climate Center network (https://www.hprcc.unl.edu). This network was explicitly designed for agricultural applications, with weather stations located within crop production areas, avoiding large urban areas or airports. Weather data have been rigorously screened to detect suspicious values following strict quality control measures. To account for spatial variation in weather, we selected three weather stations located within or nearby each region (*SI Appendix*, Fig. S1). Measured daily weather data at these stations included all the variables required for yield-potential simulation such as solar radiation and maximum and minimum temperature (T_max_ and T_min_, respectively). Finally, our simulations were based on year-specific atmospheric CO_2_ concentration, ranging from 380 ppm (2005) to 409 ppm (2018), to account for the effect of CO_2_ fertilization on maize yield potential.

We simulated yield potential for each of the 14 y (2005 to 2018) separately for each of the three weather stations selected for each region (total of 42 stations × year combinations). Some management practices, such as sowing date and cultivar maturity, can also influence yield potential via crop-cycle duration and the timing of reproductive stages in relation to the seasonal patterns in solar radiation and temperature. Because there was no single combination of sowing date and cultivar maturity that leads to a superior yield performance across all years, we simulated a total of nine different combinations of sowing date × cultivar maturity combinations for each region. These nine combinations were based on the averages for each region and two additional sowing dates (±10 d) and cultivar maturities (±2 d). These ranges covered 80% of the observed range in sowing date and cultivar maturity across farmer maize fields; all simulated combinations of sowing date × cultivar maturity existed in the real world (15). Average sowing date for each region and year corresponded to the calendar date when 50% of the total maize area was sown as reported by the Risk Management Agency (RMA-USDA). Across the 14-y time period, average sowing date was day of year (DOY) 124 (Lower Niobrara) and DOY 118 (Tri-Basin and Upper Big Blue). Average cultivar maturity for each region was retrieved from Morell et al. (56), which was consistent with our own survey data collected from a subset of maize farmers in each region. We assumed a fixed density of 8.5 plants m^−2^ for our simulations; this value represented the typical plant density used by progressive farmers to maximize yield in irrigated conditions (15, 52, 55). In all cases, our simulations assumed no water and nutrient limitations and no incidence of biotic stresses such as weeds, pathogens, and insect pests.

### Calculation of Annual Yield Gains as Driven by Climate and Technology.

For each region, the total yield gain was estimated by fitting a linear-regression model to the relationship between farm yield and time as follows:

[1] $$Ya=\beta_{0}+\beta_{1}y\mathrm{ear},$$

where Ya is farmer actual yield, $\beta_{0}$ is the intercept, and $\beta_{1}$ is the rate of yield gain (kg ha^−1^ y^−1^). We note that the model was fitted using all available field-year observations for each region. A similar approach was followed to estimate the climate-driven yield trend based on all available simulated yield-potential values for each region:

[2] $$Yc=\beta_{0}+\beta_{1}y\mathrm{ear},$$

where Yc is the climate-driven yield potential and, because simulation of yield potential over time assumed constant management and genetics, $\beta_{1}$ represents the change in yield potential solely due to climate (kg ha^−1^ y^−1^). With few exceptions, crop-yield gains are linear (3). However, much of the literature on yield gains have reported yield gains as compound exponential rates (e.g., ref. 57). To make our results comparable with these previous studies, we also expressed the total and climate-driven annual yield gain as a compound annual growth rate (% *p.a.*) to allow comparisons with estimated yield gains reported by others for environments with different yield level as follows:

[3] $$\mathrm{CAGR}=\left({\left(\frac{\mathrm{EYa}}{\mathrm{SYa}}\right)}^{\left(\frac{1}{n}\right)}-1\right)\times 100,$$

where SYa and EYa are the average farm yield at the beginning and at the end of the study period, which were derived from the fitted model following Eq. **1**, and n is the number of years included in the time period (14 in our case). Finally, the technological yield gain was estimated as the difference between total and climate-driven yield gains.

### Drivers for Climate and Technology Yield Gains.

Drivers for climate-driven yield gains were assessed by inspecting trends in accumulated solar radiation, mean daily solar radiation, and T_min_ and T_max_ over time. To do so, the crop cycle was split into three phases according to the simulated phenology: 1) vegetative (emergence to 15 d prior to silking), 2) flowering (±15 d around silking), and 3) grain-filling (from 15 d post silking to physiological maturity). Weather variables were calculated separately for each of these three phases (*SI Appendix*, Fig. S2). Time trends were investigated using linear-regression analysis based on all the simulations available for each region over time.

To identify the underpinning drivers of the technological yield gain, we conducted a survey for a subset of farmers in each region (37, 127, and 104 farmers in Lower Niobrara, Tri-Basin, and Upper Big Blue, respectively). Because each farmer in these regions manages ca. eight maize fields, we estimated that our survey was representative of ca. two-thirds of the fields included in the database and used for the analysis of yield trends. Briefly, farmers were asked to report information about key management practices for a 3-y period centered on year 2005 and 2018, separately, which corresponded to the start and end points of our time series for yield, respectively. Requested information included seeding rate, cultivar relative maturity, tillage method, proportion of maize fields in which prior crop was soybean, proportion of fields in which stover from prior crop was grazed during wintertime, and proportion of fields receiving in-season foliar fungicide and/or insecticide application. Additionally, we retrieved data on sowing date from the RMA-USDA and applied N fertilizer from the NRD database for the same two 3-y periods centered on 2005 and 2018.

Changes in management practices between the two time windows were assessed to identify candidate drivers for the technological yield gain. In the case of tillage method, fields were categorized as conventional (disk) and conservation tillage (including no till, strip till, and ridge till). Differences between time periods were evaluated using paired two-tail *t* tests, except for changes in the proportion of fields following conventional versus conservation tillage, which were assessed using χ^2^ tests. Data were assumed to follow a normal distribution; this is a reasonable assumption for samples containing more than 30 observations (as it was our case), because distribution of sample means tends to converge to a normal probability distribution function as predicted by the central limit theorem (58). Finally, we estimated the contribution of management practices to the technological yield gain in each region by summing up the expected yield gain associated with the changes in each individual practice. The latter was estimated based on relationships published in the literature for similar maize-production systems. Briefly, yield response to changes in seeding rates, tillage, crop sequence, in-season foliar fungicide and/or insecticide, and N fertilizer were retrieved from the published literature for maize crops grown in favorable environments of the US Corn Belt, mostly based on farmer data or on-farm trials conducted on irrigated fields in Nebraska (*SI Appendix*, Table S4). In the case of foliar fungicide and/or insecticide, we assumed that the yield response is the same to fungicide or insecticide alone or together. Yield response to additional N fertilizer was estimated as the product between N recovery efficiency (NRE) and N physiological efficiency (NPE). NRE and NPE were estimated for each region based on average N rate in each region by 2018 following the relationships reported by Wortmann et al. (59) for irrigated maize in Nebraska. Yield changes attributable to each agronomic practice (*SI Appendix*, Table S4) were multiplied by the associated changes in management practices between 2005 and 2018 and divided by 14 to estimate the annual yield change due to the change in each management practice. Then, we summed up the yield changes to estimate the contribution of agronomic practices to the overall technological yield gain (*SI Appendix*, Table S5). Finally, we estimated the yield gain due to genetic improvement as the difference between the overall technological yield gain and the yield gain due to agronomic management.

## Supplementary Material

Supplementary File

## Data Availability

All study data are included in the article and/or supporting information.
