# Supplementary material for: Climate and agronomy, not genetics, underpin recent maize yield gains in favorable environments
Source: Proc Natl Acad Sci U S A. 2022 Jan 18;119(4):e2113629119. doi: 10.1073/pnas.2113629119 (PMC8795556; doi:10.1073/pnas.2113629119)
Supplement: Supplementary File [file pnas.2113629119.sapp.pdf]

SI Appendix for Rizzo et al.

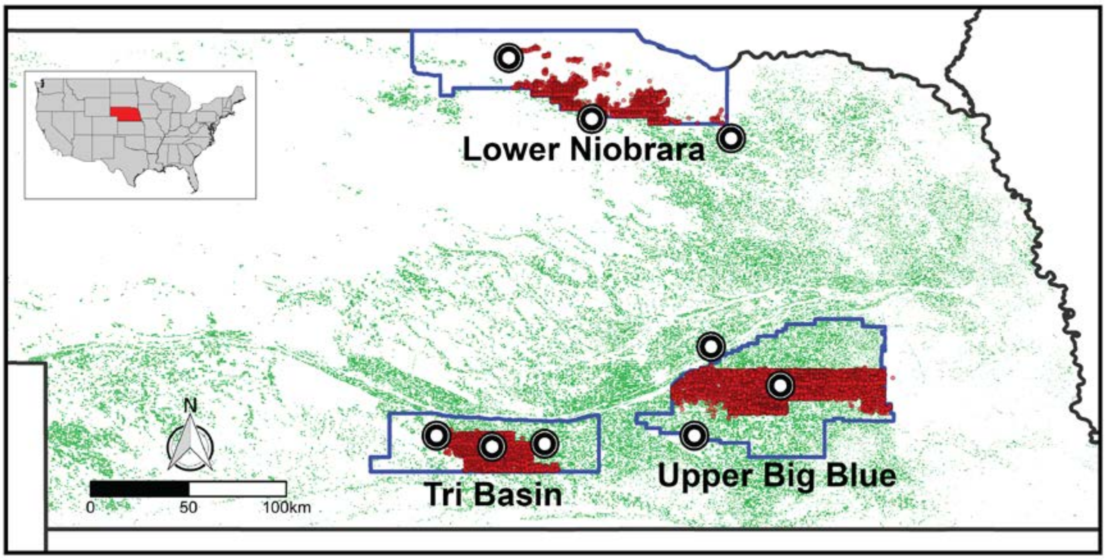

Supplementary Fig. S1. *Location of the three regions (blue polygons), farmer fields (red dots), and weather stations (circles) within Nebraska (US). Distribution of irrigated maize area is shown in green. Inset shows location of Nebraska within the conterminous US.*

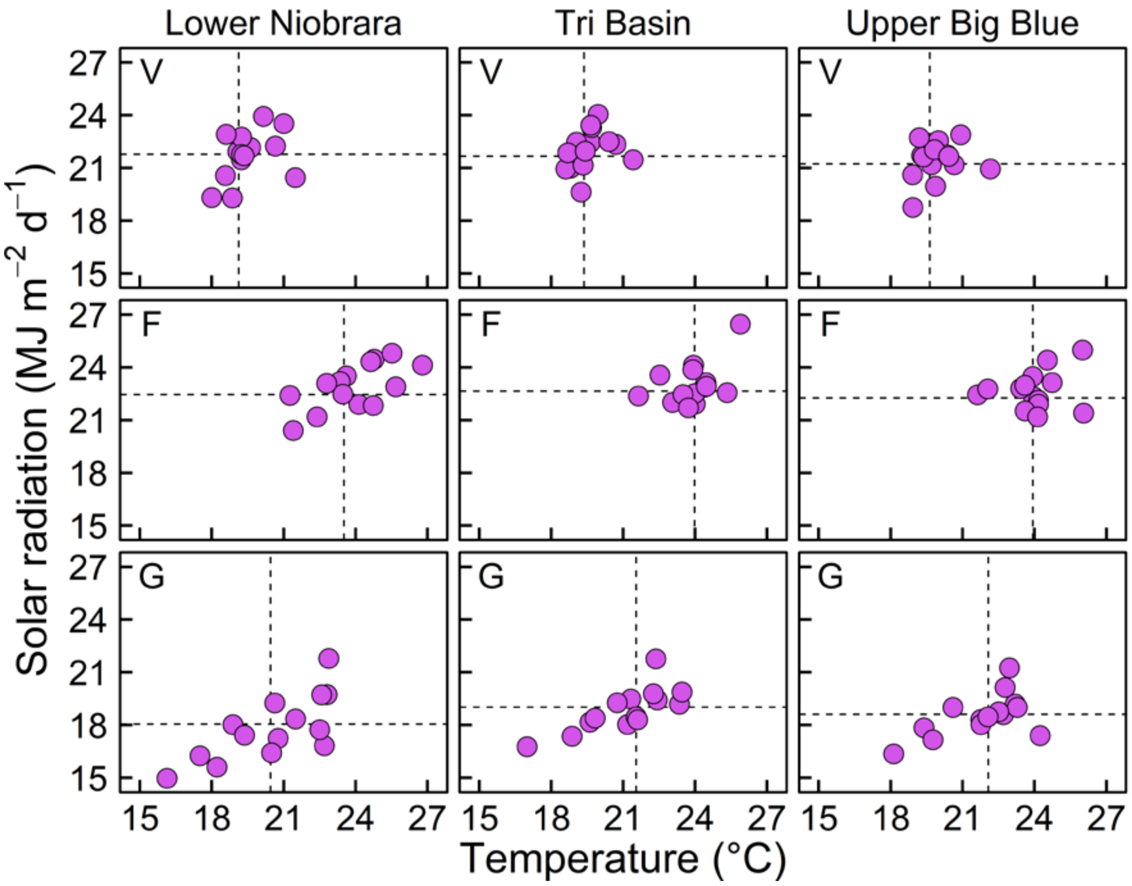

Supplementary Fig. S2. *Average solar radiation and average temperature during vegetative (V, top), flowering (F, middle), and grain filling phases (G, bottom) for each of the three regions: Lower Niobrara (left), Tri Basin (middle), and Upper Big Blue (right). Each symbol corresponds to the average value for a given year, region, and crop phase. Values are averages calculated from measured data retrieved from three weather stations located within or near each region (see Fig. 1). Dashed lines indicate long-term (1998-2018) means.*

Supplementary Table S1. *Description of key biophysical and management variables and grain yield associated with irrigated maize fields located within the three study regions in Nebraska during the 2005-2018 period. Values are means (± standard deviation). Average (2005-2018) reporting fields per year (n) is shown.*

Region	Solar radiation (MJ m^{-2} d^{-1}) and temperature (°C)[a]	Precipitation and ET_0 (mm)[a]	Dominant soil series[b]	AWHC (mm)[b]	Sowing date (DOY)[c]	Hybrid relative maturity (d)[c]	Plant density (m^{-2})[c]	Irrigation amount (mm)[d]	Applied N fertilizer (kg N ha^{-1})[d]	Maize yield (Mg ha^{-1})[d]
Lower Niobrara (n=487)	SR: 14.3±0.5<br>T: 9.3±1.0	P: 557±100<br>ET_0: 1171±136	Jansen loam & Dunday loamy sand	141±12	125±3	108±2	8.1±0.3	352±125	206±45	13.0±1.8
Tri Basin (n=1405)	SR: 15.1±0.5<br>T: 10.3±0.9	P: 524±117<br>ET_0: 1204±110	Holdrege silt loam	317±3	117±6	112±2	8.0±0.5	293±150	207±42	13.5±1.9
Upper Big Blue (n=1047)	SR: 14.4±0.4<br>T: 10.3±0.9	P: 559±120<br>ET_0: 1097±97	Hastings silt loam	285±4	118±4	113±2	8.1±0.5	277±84	204±48	13.2±1.2

[a] Annual averages for solar radiation (SR) and temperature (T) and cumulative values for precipitation (P) and grass-based Penman-Monteith-FAO reference evapotranspiration (ET_0) calculated following Allen et al. (1998) based on three weather stations located within or near each of the three regions selected for this study.
[b] Dominant soil series and average maximum available water holding capacity (AWHC; 0-1.5 m) retrieved from SSURGO soil database.
[c] Sowing date (DOY: day of year), hybrid relative maturity, and plant density retrieved from a separate database that includes *ca*. 75% of fields.
[d] Irrigation amount, applied N fertilizer, and maize grain yield retrieved from the NRD databases.

Supplementary Table S2. *Estimated total, climatic, and technological yield gains, expressed as absolute or compound annual rates, during the 2005-2018 period. The technological yield gain includes the contribution from both agronomic and genetic technologies.*

Region[†]	Compound annual growth rate			Yield gain		
	Total	Climate[‡]	Technology	Total	Climate[‡]	Technology
	% *p.a.*			kg ha^{-1} y^{-1}		
Lower Niobrara	1.27	0.78	0.49	178	109	69
Tri Basin	1.38	0.51	0.87	199	73	126
Upper Big Blue	1.29	0.60	0.69	181	84	96
Average	1.31	0.63	0.68	186	89	97

[†] The three regions included in this study account for about 43% of total USA irrigated maize production area.

[‡] The climate-driven yield potential includes the CO_2 fertilization effect, which accounts for 6% of the overall climate-driven yield gain.

Supplementary Table S3. *Changes in management practices between 2005 and 2018 based on survey data collected from a subset of farmers in three regions: Lower Niobrara (n = 37), Tri-Basin (n = 127), and Upper Big Blue (n = 104). Averages for each year and the difference between 2018 and 2005 values are shown.*

Management practice	Lower Niobrara				Tri Basin				Upper Big Blue			
	Average		Change	Yield gain	Average		Change	Yield gain	Average		Change	Yield gain
	2005	2018		kg ha^{-1} y^{-1}	2005	2018		kg ha^{-1} y^{-1}	2005	2018		kg ha^{-1} y^{-1}
Grain yield (Mg ha^{-1})	11.9	14.2	+2.3	164	12.3	14.8	+2.5	179	12.0	14.3	+2.3	164
Sowing date (DOY)	125	127	+2		117	119	+2		117	118	+1	
Seeding rate (seed m^{-2})	7.5	8.1	+0.6*	+28	7.3	8.0	+0.7*	+33	7.5	8.1	+0.6*	+28
Cultivar relative maturity (d)	108	108	nil		112	112	nil		113	113	nil	
Conservation tillage (% fields)	31	56	+25*	-6	38	88	+50*	-13	27	86	+59*	-15
Rotation with soybean (% fields)	49	54	+5	+2	49	55	+6*	+2	46	51	+5*	+2
Foliar fungicide and/or insecticide (% fields)	26	23	-3		27	65	+38*	+8	26	69	+43*	+9
Grazing prior crop stover (% fields)	58	57	-1		49	46	-3		31	34	+3	
Applied N fertilizer (kg N ha^{-1})	199	218	+19*	+29	184	223	+39*	+59	185	220	+35*	+53

DOY: day of year. Asterisks indicate statistically significant difference ($p<0.05$) using t-test or chi-square test (for variables with normal or binomial distribution, respectively).

Supplementary Table S4. *Yield benefit in irrigated maize per unit change in management practices based on data from the literature. Also shown is the source of data of each study and associated study region and associated years.*

Management practice	Yield change (kg ha^{-1}) due to unit change in management practice	Source of data and associated region and years	Source
Seeding rate (seed m^{-2})	+650	Farmers' data in Nebraska (2005-2007)	Grassini et al. (2011)
Conservation *versus* conventional tillage (% fields)	-350 [a]	Farmers' data in Nebraska (2005-2007)	Grassini et al. (2011)
Rotation with soybean *versus* continuous maize (% fields)	+500	Farmers' data in Nebraska (2005-2007)	Grassini et al. (2011)
Foliar fungicide and/or insecticide *versus* untreated (% fields)	+302	Field trials in US Corn Belt (2002-2009)	Paul et al. (2011)
Nitrogen (N) fertilizer (kg N ha^{-1})	NRE × NPE [b]	Field trials in Nebraska (2002-2004)	Wortmann et al. (2011)

[a] Only in continuous maize. No yield penalty reported when maize was rotated with soybean.

[b] Nitrogen recovery efficiency (NRE) calculated as: $y = 0.937 - 0.0018 \times N$; nitrogen physiological efficiency (NPE, kg grain kg^{-1} N uptake) calculated as $y = 31.39 + 37.75 \times 0.993^{N}$.

Supplementary Table S5. *Contribution of adoption of improved agronomic practices and genetic yield potential to the overall technological yield gain, expressed as absolute or compound annual rates.*

Region[†]	Compound annual growth rate			Yield gain		
	Technology	Agronomic	Genetics	Technology	Agronomic	Genetics
	% *p.a.*			kg ha^{-1} y^{-1}		
Lower Niobrara	0.49	0.37	0.12	69	53	16
Tri Basin	0.87	0.61	0.26	126	89	37
Upper Big Blue	0.69	0.55	0.14	96	77	19
Average	0.68	0.51	0.17	97	73	24
